# Characterization of Two Distinct Nucleosome Remodeling and Deacetylase (NuRD) Complex Assemblies in Embryonic Stem Cells* [Fn FN2]

**DOI:** 10.1074/mcp.M115.053207

**Published:** 2015-12-29

**Authors:** Daniel Bode, Lu Yu, Peri Tate, Mercedes Pardo, Jyoti Choudhary

**Affiliations:** From the ‡Proteomic Mass Spectrometry, Wellcome Trust Sanger Institute, Wellcome Trust Genome Campus, Hinxton CB10 1SA, UK;; §Stem Cell Engineering, Wellcome Trust Sanger Institute, Wellcome Trust Genome Campus, Hinxton CB10 1SA, UK

## Abstract

Pluripotency and self-renewal, the defining properties of embryonic stem cells, are brought about by transcriptional programs involving an intricate network of transcription factors and chromatin remodeling complexes. The Nucleosome Remodeling and Deacetylase (NuRD) complex plays a crucial and dynamic role in the regulation of stemness and differentiation. Several NuRD-associated factors have been reported but how they are organized has not been investigated in detail. Here, we have combined affinity purification and blue native polyacrylamide gel electrophoresis followed by protein identification by mass spectrometry and protein correlation profiling to characterize the topology of the NuRD complex. Our data show that in mouse embryonic stem cells the NuRD complex is present as two distinct assemblies of differing topology with different binding partners. Cell cycle regulator Cdk2ap1 and transcription factor Sall4 associate only with the higher mass NuRD assembly. We further establish that only isoform Sall4a, and not Sall4b, associates with NuRD. By contrast, Suz12, a component of the PRC2 Polycomb repressor complex, associates with the lower mass entity. In addition, we identify and validate a novel NuRD-associated protein, Wdr5, a regulatory subunit of the MLL histone methyltransferase complex, which associates with both NuRD entities. Bioinformatic analyses of published target gene sets of these chromatin binding proteins are in agreement with these structural observations. In summary, this study provides an interesting insight into mechanistic aspects of NuRD function in stem cell biology. The relevance of our work has broader implications because of the ubiquitous nature of the NuRD complex. The strategy described here can be more broadly applicable to investigate the topology of the multiple complexes an individual protein can participate in.

Chromatin remodeling provides an essential regulatory mechanism for pluripotency and differentiation in stem cell biology (1). Addition or removal of modifications to histone tails enable alteration to the condensation state of chromatin structures, significantly altering promoter accessibility for transcription. In embryonic stem cells, self-renewal genes are constitutively expressed and to initiate lineage commitment and advance embryonic development, they are gradually silenced (2). Key pluripotency transcription factors Nanog and Oct4 have been shown to be transcriptionally repressed by the Nucleosome Remodeling and Deacetylase (NuRD)^1^ complex (3). NuRD is further involved in the deacetylation of H3Lys^9^ac at enhancers of pluripotency genes, enabling Lsd1-mediated removal of the H3K^4^me1 activating mark (1). Furthermore, the NuRD complex plays an important role in maintaining the bivalent state of differentiation genes in ESCs. These loci are kept repressed but in a so-called “poised” state, ready for rapid expression upon differentiation signaling, through the simultaneous presence of H3K^4^me3 at promoter regions and H3K^27^me3 at the open reading frame (4). Both bivalent promoter regions and active loci have been identified as targets of the NuRD complex. NuRD catalyzes H3K^27^ deacetylation and recruits the Polycomb repressive complex 2 (PRC2). In turn, PRC2 trimethylates H3K^27^, leading to transcriptional repression (5–7).

The hetero-oligomeric NuRD complex contains two catalytic subunits, Chd3/4 and Hdac1/2. Chd3 and Chd4 catalyze ATPase-mediated nucleosomal sliding, whereas Hdac1 and Hdac2 deacetylate histone proteins. Other subunits include Mbd2/3, Mta1/2/3, Rbbp4/7, and Gatad2a/b, which exhibit regulatory and scaffolding functions (8–11). Mbd3 is essential for the structural integrity of NuRD and targets the complex to promoters with transcriptional activation marks in murine embryonic stem cells (6, 12). Gatad2a and Gatad2b, also known as p66, assist in the assembly of the NuRD complex and in the binding to histone tails (13). Rbbp4 and Rbbp7 are chaperones aiding in the catalytic function of the NuRD complex. The coregulators of the Mta family mediate the interaction with transcription factors to recruit the repressive NuRD function to specific target loci (10). Transcription factors and other chromatin binding factors are also able to associate with NuRD (6). Despite some studies addressing the stoichiometry of NuRD complex subunits (14, 15), how the various interacting partners are organized has not been explored in detail.

To dissect the molecular function of multiprotein complexes, it is vital to develop reliable procedures for resolving the interactome. Affinity purification coupled with tandem mass spectrometry (AP-MS) has become one of the most frequently applied methods for identifying protein–protein interactions. However, a simple purification of a bait protein and its associated interactors yields a one-dimensional list of interacting proteins, which is not representative of how these proteins associate with each other (16). Proteins often appear in several distinct complexes with different functions depending on the association of different binding partners. Hence, to fully exploit the potential of AP-MS it is crucial to resolve individual protein complexes. Several strategies have been used to derive topological information and/or distinguish between protein assemblies sharing subunits. To elucidate subcomplex variation all assumed subunits can be iteratively analyzed by AP-MS (17). Such approach is experimentally and analytically time consuming. Protein interactions can be cross-linked to widen the scope of the topological analysis and identify direct interactions (18). However, this strategy requires intense computational analysis, thus increasing the complexity of data interpretation. Biochemical fractionation allows isolation of a multiprotein complex of interest and thus enables direct determination of the topology. Frequently used techniques include gel filtration, density gradient centrifugation and ion exchange chromatography. All these were recently combined with LC-MS/MS to describe protein complexes without prior purification (19–22).

To widen the scope of fractionation techniques, we have used Blue Native polyacrylamide gel electrophoresis (BN-PAGE) and applied it to affinity-purified soluble complexes. In contrast to the most widely used SDS-PAGE, BN-PAGE does not require denaturation of the samples, because the detergent is replaced by the anionic Coomassie G-250 dye. CBB induces a charge shift by binding to the multimer's surface without altering the tertiary and quaternary structures (23–25), allowing migration of native complexes including hydrophobic protein subunits through a gel (26, 27). Previous studies using BN-PAGE focused mostly on resolving mitochondrial complexes (28, 29) and other membrane complexes (30), with also scattered studies on nuclear lysates (31) and whole cell lysates (32). In the last five years a few studies have combined BN-PAGE with quantitative MS and protein correlation profiling for the identification of protein complexes, mainly in mitochondria and proteasome (26, 33–36).

In this study, we combined AP with BN-PAGE followed by protein identification by LC-MS/MS and protein correlation profiling to characterize affinity-purified chromatin remodeling complexes from mouse embryonic stem cells. We show that in murine ESCs the NuRD complex exists as two distinct entities and show that only one of them interacts with the transcriptional repressor Sall4, specifically with isoform Sall4a. We also identify a novel interaction of NuRD with the MLL complex regulatory subunit Wdr5.

## EXPERIMENTAL PROCEDURES

### 

#### 

##### Cell Culture

The feeder independent 129ola strain derived ES cell line E14Tg2a was used for gene targeting. Cells were cultured in GMEM (Sigma-Aldrich, St. Louis, MO) in the presence of leukemia inhibitory factor (Life Technologies, Waltham, MA) according to standard procedures. Large scale cell culture was carried out by StemCell Technologies Inc. (Cambridge, UK).

##### Affinity Purification

Large scale affinity purification of Mta2-FTAP was performed essentially as described previously (37) with the following modifications. The lysis buffer contained 450 mm NaCl and 0.2% Nonidet P-40. The clarified lysate was diluted 1:3 to 150 mm NaCl and 0.1% Nonidet P-40 before incubation with the beads. Affinity-purified proteins were eluted from the beads by incubation in 200 μg/ml 3× FLAG peptide in elution buffer (10 mm Tris-HCl, 150 mm NaCl, 0.02% Nonidet P-40, 200 mm ε-aminocaproic acid). The eluate was concentrated in Vivaspin 500 centrifugal device (PES, 5000 Da cut-off, GE Healthcare, Little Chalfont, UK) prior to BN-PAGE.

##### Blue Native Polyacrylamide Gel Electrophoresis

The Native PAGE system from Life Technologies was used. Samples were mixed with 4×Native PAGE Sample Buffer (Life Technologies) and Native PAGE 0.5% G-250 Sample Additive (Life Technologies) was added to a final concentration of 0.05%. The electrophoresis was performed using Native PAGE 3–12% Bis-Tris gels (Life Technologies). NativeMark Unstained Protein Standard (20kDa–1.2MDa, Life Technologies) was used as a molecular weight marker. Electrophoretic buffers were prepared and used according to the manufacturer's protocol (Life Technologies). After one third of the migration distance, the cathode buffer was replaced by Light Blue Cathode Buffer. Coomassie staining was performed as described previously (37).

##### Size Exclusion Chromatography

A mouse ESC whole cell lysate was prepared as described previously (37) and then treated with benzonase (Sigma-Aldrich, 90% purity, 1 μl per ml lysate). The lysate (2.5 mg total protein) was injected on a Superdex 200 10/300 GL (GE Healthcare) column equilibrated with diluted lysis buffer (50 mm Tris pH 8, 150 mm NaCl, 0.1% Nonidet P-40) using an AKTA Pure FPLC system (GE Healthcare). The flow rate was set at 0.5 ml/min and 28–500 μl fractions were collected in 96-deep-well plates. UV absorbance readings were acquired at 260 nm, 280 nm, and 214 nm. The fractions spanning the protein-containing part of the chromatograph were concentrated in Vivaspin 500 centrifugal devices (PES, 5000 Da cut-off) prior to PAGE.

##### Western Blotting

For denaturing PAGE, samples were separated in NuPAGE Bis-Tris 4–12% gels (Life Technologies) using MOPS running buffer according to manufacturer's instructions. Gels were transferred to nitrocellulose membranes (Bio-Rad, Hercules, CA) using XCell Transfer Kit and Transfer Buffer (Life Technologies) according to manufacturer's instructions. BN-PAGE gels were first incubated in 8% acetic acid prior to transfer to PVDF membranes (Life Technologies). Protein detection was performed using the following antibodies: FLAG (M2-peroxidase labeled, A8592, Sigma-Aldrich), Sall4 (ab29112, Abcam, Cambridge, UK), Chd4 (A301–081A, Bethyl Laboratories Inc., Montgomery, TX), Hdac2 (sc-7899, Santa Cruz, Biotechnology Inc., Dallas, TX), Wdr5 (A302–430A, Bethyl Laboratories Inc.), Wdr5 (A302–429A, Bethyl Laboratories Inc.), and Rbbp4 (ab1765, Abcam).

##### LC-MS/MS Analysis

BN gels were excised into 48 identical (1.5 mm × 5 mm) slices and processed as described previously (37). Peptides were redissolved in 0.5% formic acid and analyzed with online nanoLC-MS/MS on an Orbitrap Velos mass spectrometer (ThermoFisher Scientific, Waltham, MA) coupled with an Ultimate 3000 Nano/Capillary LC System (Dionex, Sunnyvale, CA). Samples were first loaded and desalted on a nanotrap (100 μm id × 2 cm) at 10 μl/min with 0.1% formic acid for 10 min and then separated on an analytical column (75 μm id × 15 cm) (both PepMap C18, Dionex) over a 30 min linear gradient of 4–28% CH_3_CN/0.1% formic acid at 300 nL/min. The Orbitrap Velos was operated in standard data-dependent acquisition. The survey scans (*m*/*z* 380–1600) were acquired in the Orbitrap at a resolution of 30,000 at m/z 400, and one microscan was acquired per spectrum. The ten most abundant multiply charged ions with a minimal intensity of 3000 counts were subject to MS/MS in the linear ion trap at an isolation width of 2 Th. Dynamic exclusion width was set at ± 10 ppm for 45 s. The automatic gain control target value was regulated at 1 × 10e^6^ for the Orbitrap and 5000 for the ion trap, with maximum injection time at 150 ms for Orbitrap and 100 ms for the ion trap, respectively.

##### MS Data Analysis

Spectral analysis for protein identification and quantitation was performed using MaxQuant (version 1.5.1.2) (38, 39). The Mouse Uniprot reference proteome (January 2014; containing 51,159 entries) was used for the database search. Cysteine carbamidomethylation was set as fixed modification and methionine oxidation and acetylation (N-terminal) were set as variable modifications. Trypsin was specified as protease, with a maximum of two missed cleavage sites allowed. Precursor mass tolerance was set at 4.5 ppm and fragment mass tolerance was set at 0.5 Da. The maximum false discovery rate was set at 0.01 for both peptide and protein identifications, based on hits to a reversed decoy database. MS data from the BN-PAGE fractions were processed in batch as separate experiments to derive independent quantification values for each fraction. The minimum ratio count was set to one, unique and razor peptides were used for quantification, and iBAQ values were computed. The proteinGroups results file was processed in Perseus (Version 1.5.0.31) for removal of contaminants, reverse IDs, and proteins only identified by site. We used five replicates of a nonrelevant FTAP2-tagged control (P. Tate, manuscript in preparation) affinity purification to draw a list of common nonspecific binding proteins in FLAG affinity purifications (data not shown). Data visualization and further processing was performed using scripts developed in R (Version 2.15.3). Hierarchical clustering was carried out using the average distance in a Manhattan distance matrix and the resulting clustering was visualized in a heat-map across all BN-PAGE fractions.

The raw MS data has been submitted to the ProteomeXchange Consortium via the PRIDE partner repository with the data set identifier PXD002452.

##### Bioinformatics Analyses of ChIP-seq Data Sets

Chromatin immunoprecipitation followed by deep sequencing (ChIP-Seq) data sets for Chd4, Hdac1, Hdac2, Wdr5, Sall4, and Suz12 were retrieved from published studies (GEO repository accession numbers: GSE12482, GSE19588, GSE27970, GSE27844; ArrayExpress database accession numbers: E-MTAB-888, E-MTAB-889). All these data sets were derived from mouse embryonic stem cells. GO term enrichment analysis was performed using DAVID v6.7 (40, 41).

## RESULTS

### 

#### 

##### Blue Native Polyacrylamide Gel Electrophoresis Can Resolve the NuRD Complex

**Fig. 1.**
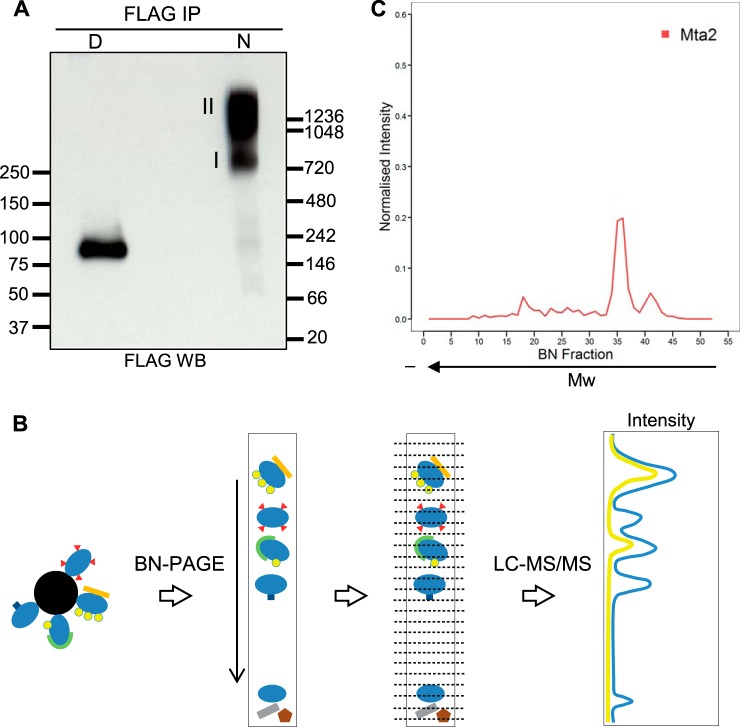
**Reproducible separation of Mta2-containing complexes by Blue Native PAGE.** *A*, FLAG Western blotting of FLAG immunoprecipitates from Mta2-FTAP cells after blue native PAGE separation. The immunoprecipitate was divided in two aliquots, one was denatured prior to BN-PAGE (D) and the other one was left native (N). Molecular weight markers are indicated in kDa. *B*, Schematic workflow of the experimental set-up used in this study. Affinity-purified protein complexes were separated by BN-PAGE. The gel lane containing the complexes was excised into 48 equal fractions from bottom to top, which were then analyzed individually by LC-MS/MS. The quantitation data from each fraction was used to generate a migration profile for each protein across the BN-PAGE separation length. *C*, Migration profile of Mta2 across the separation length of the gel.

To effectively isolate the NuRD complex in native conditions, we used a mouse E14 ESC line where Mta2, a core subunit of the NuRD complex, was epitope-tagged at the endogenous locus using a gene targeting approach. A detailed description of the tagging procedure is reported elsewhere (P. Tate, manuscript in preparation). In brief, a high efficiency gene targeting cassette containing the FTAP2 tag, a slight modification on the FTAP tag (37) (see Supplemental Fig. 1) was used to construct a targeting vector from a mouse genomic BAC by recombineering (42), which when transfected into mouse ESCs lead to introduction of the tag at the carboxy-terminus of the endogenous *Mta2* open reading frame. This tagging approach can be applied in a high-throughput fashion, and we have shown that tagged ESC can be used to generate tagged mice (43). We purified Mta2 and associated proteins by affinity capture using an anti-FLAG antibody, eluted them in native conditions by competition with FLAG peptide, and analyzed the eluate by blue native PAGE. To test the separation and the integrity of Mta2-containing complexes we first aimed to determine the migration pattern of the NuRD complex by Western blotting after BN-PAGE. We probed for Mta2 in the native sample and also in the denatured eluate. Although the denatured sample yielded a single band at the expected monomeric molecular weight, in the native sample Mta2 migrated as a smear above 720 kDa, in agreement with the predicted molecular weight of the NuRD complex (assuming a single molecule of each known subunit) (Fig. 1*A*). Two distinct regions could be distinguished within the smear, suggesting there might be different Mta2-containing assemblies. Monomeric Mta2 was also observed as a very faint band migrating at the same size as denatured Mta2. We then probed for other subunits of NuRD, namely Hdac1 and Hdac2; however, detection of these subunits in the native sample was unsuccessful (data not shown).

To characterize native Mta2-containing protein complexes, the gel lane was excised in 48 equal fractions from bottom to top (Fig. 1*B*). Following in-gel digestion, we analyzed each fraction by LC-MS/MS. MS data for each fraction was analyzed independently with MaxQuant to derive protein identification and quantitation (supplemental Table S1). We then generated migration profiles for all identified proteins by plotting intensities for all fractions across the separation length of the gel (Fig. 1*B*). To enhance profile comparison between proteins, the fraction intensity of each protein was normalized against the protein's total intensity across the whole profile. We performed two biological replicates of this experiment to ensure reproducibility.

We first focused on Mta2 and other subunits of the NuRD complex. The migration profile of Mta2 showed two distinct peaks in the region of 1–1.2 MDa, in agreement with the Western blot results (Fig. 1*C*). To determine the reproducibility, we compared the migration profiles of Mta2 in both replicates. Because cutting differences precluded alignment of the profiles by fraction number, we aligned them using the fraction where Mta2 was found at highest intensity (supplemental Fig. S2*A*). We observed identical separation pattern into two distinct complex entities in the two replicates. To ensure that the interactions and patterns observed are not DNA-mediated we repeated the above procedure including benzonase, a DNA and RNA nuclease, during the affinity purification. We carried out two benzonase-treated replicates. We again observed the same separation pattern, closely correlated to experiments performed without benzonase (supplemental Figs. 2*B* and S3). Collectively this data shows that the BN-PAGE-MS approach is reproducible and can be used to resolve affinity-purified Mta2-containing complexes.

##### The NuRD Complex Forms Two Distinct Entities

**Fig. 2.**
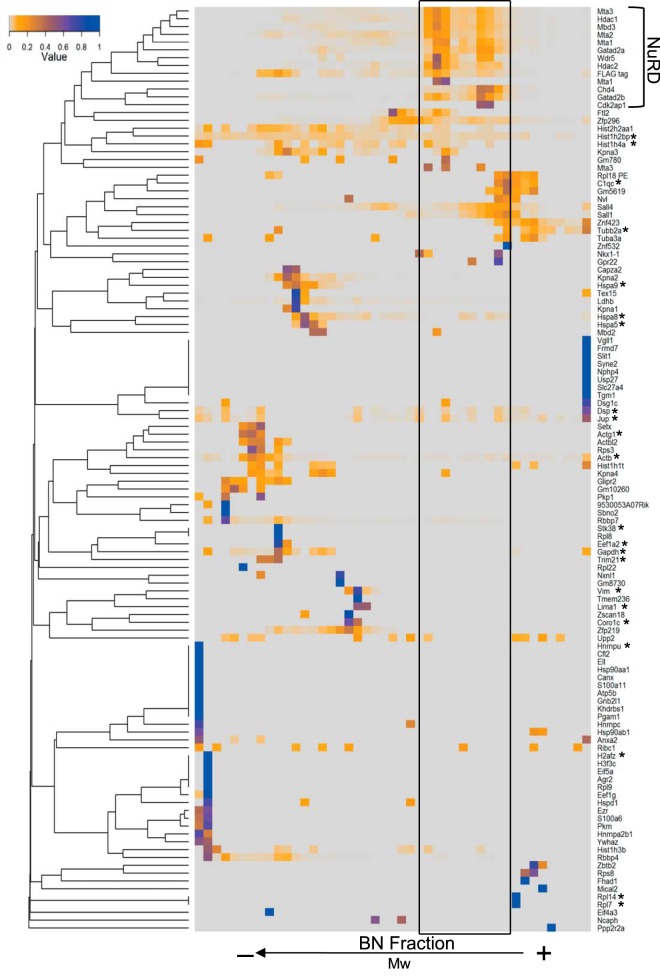
**Hierarchical clustering of all identified Mta2-associated proteins.** All identified binding proteins were clustered according to the similarity of their migration profiles. The resulting dendrogram and the associated intensity peak distribution are visualized in a heat-map. The NuRD complex cluster is highlighted and the resulting region of interest is indicated. Nonspecific binding proteins, based on five replicate control affinity purification experiments, are labeled with an asterisk.

We next looked at core NuRD subunits. Proteins interacting within complexes would be expected to have similar BN-PAGE migration profiles and hence protein correlation profiling can be applied to identify interacting proteins (22, 26). To compare the protein migration profiles in a systematic manner we computed the hierarchical clustering of all identified proteins across all BN fractions and visualized the clustered regions in a heat-map (Fig. 2). This analysis was performed on a benzonase-treated data set.

**Fig. 3.**
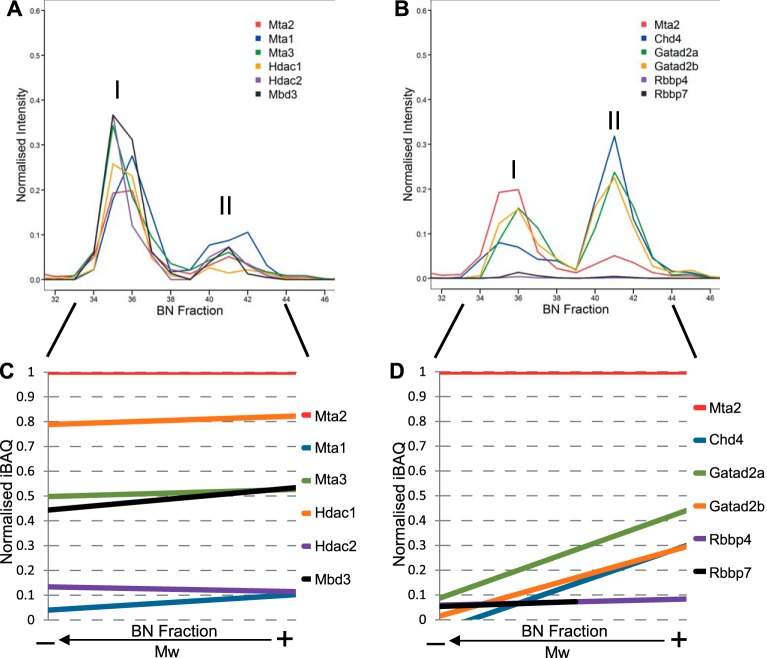
**The core NuRD complex subunits associate in two distinct entities.** *A*, Migration profiles for core NuRD subunits Mta1/2/3, Hdac1/2, and Mbd3 across the BN-PAGE separation length. *B*, Migration profiles for core NuRD subunits Chd4, Gatad2a/b, and Rbbp4/7 across the BN-PAGE separation length. Only fractions from the range of interest (32–46) in a representative experiment are displayed. *C*, Linear regression plot of normalized iBAQ values relative to Mta2 for Mta1/2/3, Hdac1/2, and Mbd3 outlining their similarity to the migration profile of Mta2. Intensity-based total quantification (iBAQ) values were computed for each identified protein in each fraction. Values were normalized against the bait Mta2 iBAQ value. *D*, Linear regression plot of normalized iBAQ values relative to Mta2 for Chd4, Gatad2a/b, and Rbbp4/7. Chd4 and Gatad2a/b show steep ascending trends indicative of their different contribution to the two NuRD entities. The linear regressions in *C* and *D* were calculated only from fractions within the peak limits from replicates performed with benzonase (fractions 26–36).

As expected we observed tight clustering of all other known core NuRD subunits, both catalytic and regulatory, with their profiles displaying two peaks coincident with those of Mta2 (Fig. 2). The migration profiles of most subunits, Mbd3, Hdac1/2, Mta1/2/3, and Rbbp4/7, correlated well to the bait protein Mta2 in abundance, with higher intensity in the low-mass peak, which we termed NuRD I (Fig. 3*A*, 3*B*). In contrast, Chd4, Gatad2a, and Gatad2b displayed increased intensity in the high-mass peak, termed NuRD II (Fig. 3*B*).

To compare the relative amounts of NuRD subunits in the fractions we next computed their intensity based absolute quantification index (iBAQ) (44) in each fraction separately and normalized to the corresponding Mta2 iBAQ value in that fraction. We plotted the correlation of each NuRD subunit to Mta2 (supplemental Fig. S4) and applied linear regression within the previously determined NuRD migration region (Fig. 3*C*, 3*D*). A horizontal trend line is indicative of high correlation to the profile of Mta2 in quantitative terms and reveals that the subunit is in the same relative amount to Mta2 throughout the separation. NuRD core subunits Mta1, Mta3, Hdac1, Hdac2, Mbd3, Rbbp4, and Rbbp7 showed such horizontal trend, suggesting similar distribution among both NuRD complex variants (Fig. 3*C*, 3*D*). Chd4, Gatad2a, and Gatad2b displayed an ascending slope (Fig. 3*D*), suggesting that these subunits are over-represented in the high-mass variant. The profiles of Gatad2a and Gatad2b (p66α/β respectively) correlated closely as expected based on their high sequence and functional homology (10). iBAQ values in the NuRD II assembly are in agreement with previously reported core NuRD stoichiometry (15), but NuRD I does not conform to this model.

In summary, we conclude that the BN-PAGE approach is reproducible and suitable for resolving soluble multiprotein complexes after affinity purification. Our results suggest that in mESCs the NuRD complex is present as two distinct assemblies with varying topology and subunit composition, with Chd4 and p66 being more abundant in the high-mass entity.

##### NuRD Associates with the MLL Methyltransferase Complex Regulatory Subunit Wdr5

**Fig. 4.**
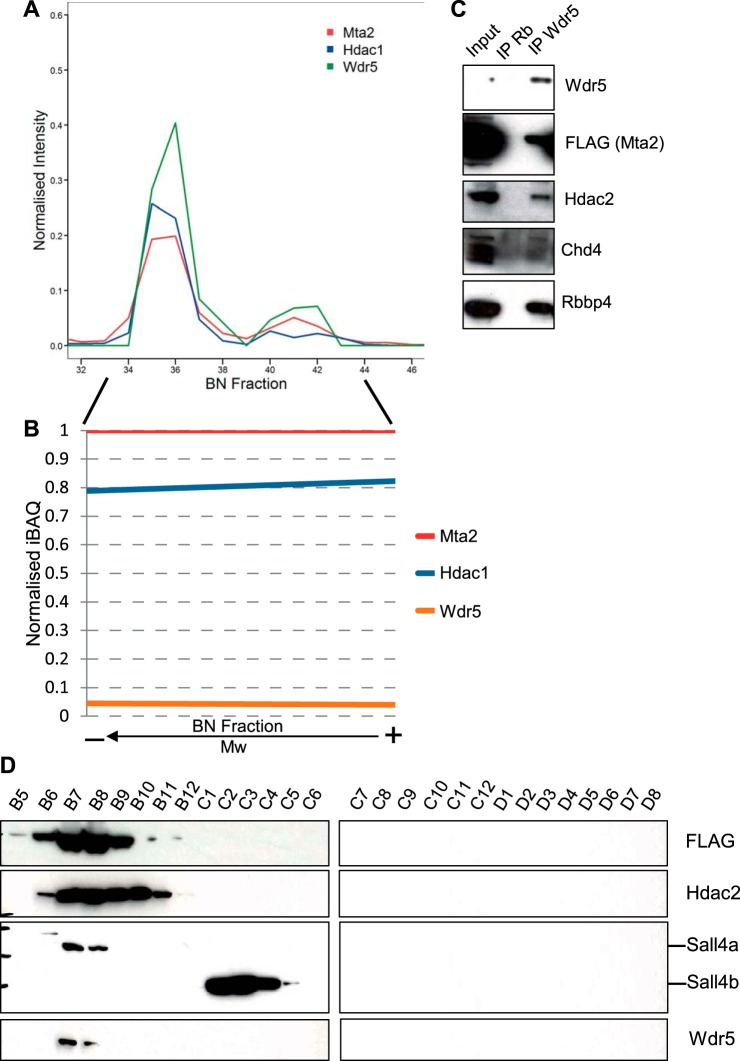
**NuRD associates with the MLL methyltransferase complex regulatory subunit Wdr5.** *A*, BN-PAGE migration profile of Wdr5 showing correlation to the NuRD reference profiles of Mta2 and Hdac1. *B*, Linear regression plot of normalized iBAQ relative to Mta2 for Wdr5, illustrating the comigration trend. *C*, Western blotting for NuRD subunits Mta2 (FLAG), Hdac2, Chd4, and Rbbp4 following Wdr5 IP. *D*, An ESC whole cell lysate was subject to size exclusion chromatography. Twenty-eight fractions were collected (3B5–3D8) and analyzed by Western blotting for Mta2-FLAG, Hdac2, Sall4, and Wdr5.

To identify other novel putative interacting proteins we studied the protein clusters in detail and looked for proteins with profiles clustering close to NuRD. Interestingly, we observed a very strong correlation between the migration profiles of Wdr5 and NuRD subunits, as reflected by PCP and the overlaying of migration profiles (Figs. 2, 4*A*). Wdr5 is the regulatory subunit of the MLL methyltransferase complex (45). The profile of Wdr5 showed two peaks corresponding to both NuRD entities, with intensities correlating those of the core subunits (Fig. 4*A*). We generated the iBAQ-derived linear regression for Wdr5 to correlate amount of Wdr5 to that of Mta2 across fractions and found it to have a horizontal slope, in a similar way to Hdac1/2, Mbd3, and Rbbp4/7 subunits (Fig. 4*B*).

We next went on to confirm such an interaction. We immunoprecipitated Wdr5 from benzonase-treated lysates and probed the immunoprecipitate for subunits of NuRD (Fig. 4*C*). The catalytic subunits Chd4 and Hdac2 co-immunoprecipitated with Wdr5, and so did Mta2 and Rbbp4.

Finally, we separated an ESC whole cell lysate by SEC (supplemental Fig. S5), collected 28 elution fractions and analyzed them by Western blotting for subunits of NuRD and Wdr5 (Fig. 4*D*). Detection of FTAP-tagged Mta2 and Hdac2 marked the elution spectrum of the NuRD complex. We observed co-elution of Wdr5 with NuRD subunits in several fractions (Fig. 4*D*).

In brief, these results indicate that Wdr5 associates with the NuRD complex in a stable and DNA-independent manner. The interaction between Wdr5 and NuRD is very similar to that between core NuRD subunits themselves, in that it is able to withstand high salt extraction, affinity purification and BN-PAGE, suggesting it is reasonably strong.

##### The NuRD II Entity Associates Exclusively with Sall-like Protein 4 Isoform A

We next explored the difference between NuRD I and II assemblies by identifying migration profiles with peaks correlating with only one of the two NuRD peaks in the hierarchical clustering.

**Fig. 5.**
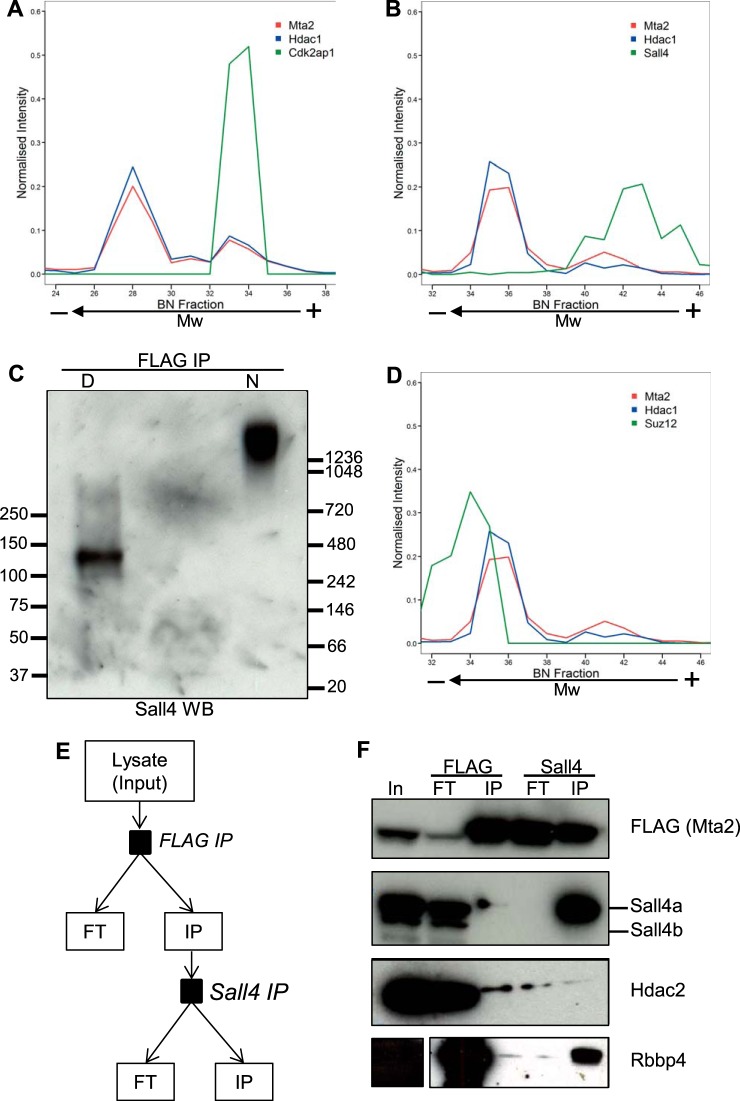
**Sall4 associates with the high-mass, but not with the low-mass NuRD entity.** *A*, Migration profiles for Mta2, Hdac1, (NuRD reference profiles) and Cdk2ap1. *B*, Migration profiles for Mta2, Hdac1, and Sall4, which outline comigration of Sall4 with the high-mass NuRD entity. Mta2 and Hdac1 provide a reference profile of the NuRD complex. *C*, Sall4 Western blotting of FLAG immunoprecipitates from Mta2-FTAP cells after BN-PAGE separation. The immunoprecipiate was divided in two aliquots of which one was denatured (D) and the other left native (N) prior to BN-PAGE. Molecular weight markers are indicated in kDa. *D*, Suz12 migration profile in the NuRD region. Mta2 and Hdac1 are references for the NuRD migration profile. *E*, Schematic diagram of the serial immunoprecipitation procedure. FLAG was isolated from the whole cell lysate. The immunoprecipitate provided the input for the subsequent Sall4 purification. The lysate, both immunoprecipitates (IP) and both flow-throughs (FT) were subject to Western blotting. *F*, Successive FLAG (Mta2) and Sall4 IPs were performed, first on a whole cell lysate from Mta2-FTAP cells and subsequently on the eluate from the first IP. The input lysate, immunoprecipitates (IP) and flow-throughs (FT) were probed for FLAG, Sall4, Hdac2, and Rbbp4.

The migration profile of cell cycle regulator Cdk2ap1 (46) clustered closely with those of NuRD subunits (Fig. 2). Cdk2ap1 has previously been shown to associate with NuRD subunits and to have a role in ESC pluripotency (47–49). Comparison of these profiles revealed that Cdk2ap1 comigrates with the NuRD II assembly but not with NuRD I (Fig. 5*A*).

Transcriptional repressor Sall4 has been previously reported to interact with the NuRD complex in embryonic stem cells (14, 50). In our lab, we have also consistently identified Sall4 as a NuRD-associated protein and, *vice versa*, NuRD subunits as Sall4-interacting proteins (data not shown). Sall4's migration profile also displayed a distinct peak that correlated well with the high-mass NuRD II assembly and extended beyond it (Fig. 5*B*). In contrast, a peak corresponding to the low-mass NuRD I assembly was not observed. This result mirrored the Sall4 immunodetection in samples separated by BN-PAGE. In the denatured sample, Sall4 was observed as two bands at the expected monomeric molecular weights, corresponding to isoforms A and B. In the native sample, the Sall4 signal appeared as a smear above 1200 kDa, coincident with the higher Mw part of the Mta2 signal (Fig. 5*C*). This suggests that only the high-mass NuRD entity, but not the low-mass, associates with Sall4 and Cdk2ap1. In contrast, the PRC2 core regulatory subunit Suz12, partially correlated with the low-mass assembly and was absent from the high-mass region (Fig. 5*D*).

To distinguish between the two NuRD assemblies and confirm the Sall4 observation, we separated the two forms of NuRD using a different approach. We carried out successive Mta2 (via FLAG) and Sall4 immunoprecipitations and probed all inputs, flow-throughs, and eluates for core subunits of NuRD (Mta2, Hdac2, and Rbbp4) and Sall4. We first immunoprecipitated Mta2-FTAP with anti-FLAG antibody from whole cell lysates, eluted the bound material in native conditions by competition with FLAG peptide, and then immunoprecipitated Sall4 from the eluted material until depleted (Fig. 5*E*). NuRD bait Mta2-FTAP was successfully immunoprecipitated from whole cell lysates by anti-FLAG, and Hdac2, Rbbp4, and Sall4 co-immunoprecipitated with it (Fig. 5*F*). After anti-Sall4 immunoprecipitation, Sall4 was depleted from the FLAG-eluted material and found only in the Sall4 immunoprecipitate. By contrast Mta2, Hdac2, and Rbbp4 were found in both the Sall4 immunoprecipitate and the flow-through. These results confirm that Sall4 interacts with the NuRD complex in mESCs and, furthermore indicate that only a fraction of NuRD interacts with Sall4, thus validating the observations from the BN-PAGE experiment. Although we did not detect Cdk2ap1 in the second immunoprecipitation step by Western blotting, mass spectrometry analysis of Sall4 IP eluates conclusively identified Cdk2ap1 as a Sall4-binding protein (data not shown).

Sall4 exists in two isoforms (Sall4a and Sall4b) with distinct molecular weights, both of which can form homodimers or a Sall4a/b heterodimer. The Sall4 antibody we used was raised against a region present in both isoforms. Interestingly, only Sall4a but not Sall4b co-immunoprecipitated with NuRD (Fig. 5*F*). To further investigate the isoform specificity of the NuRD-Sall4 interaction, we separated an ESC whole cell lysate by size exclusion chromatography, and analyzed elution fractions by Western blotting (Fig. 4*D*). Detection of FTAP-tagged Mta2 and Hdac2 marked the elution spectrum of the NuRD complex. Mta2-FTAP eluted in fractions B5-B11, whereas Hdac2 was observed in fractions B6 to B11. The molecular weight difference between Sall4a and Sall4b allowed distinguishing between both isoforms. Detection of Sall4 in SEC fractions showed that only Sall4a co-elutes with the NuRD complex, whereas Sall4b eluted at a lower molecular weight (Fig. 4*D*). Prolonged exposure resulted in appearance of a reduced Sall4a signal in several of the Sall4b-containing fractions (data not shown). Consequently, this suggests that the previously described Sall4-targeted repression via association with the NuRD complex is primarily facilitated by isoform b.

In summary, our results confirm previously reported interactions of NuRD with Cdk2ap1 and Sall4 and further establish that this interaction is specific to isoform Sall4a. Moreover, we show that only a fraction of total NuRD associates with Sall4 and Cdk2ap1.

##### Co-occupancy of Target Genes by NuRD, Sall4, Wdr5, and Suz12

**Fig. 6.**
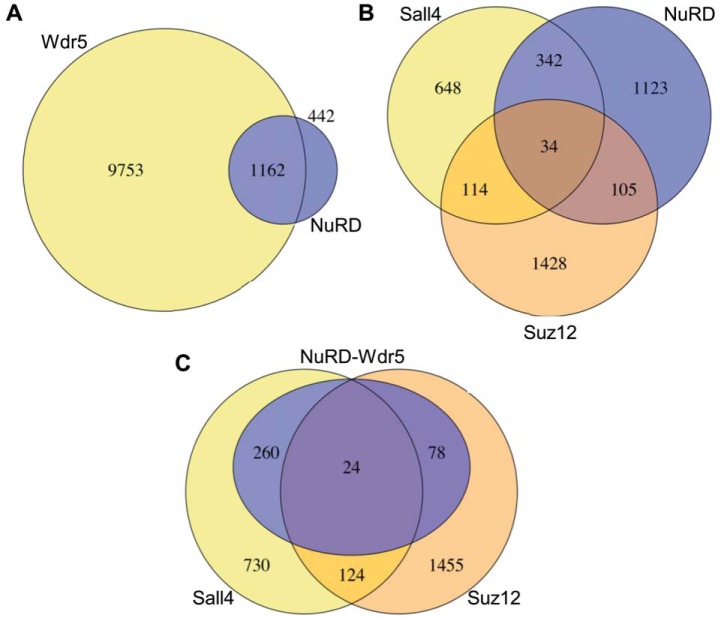
**Co-occupancy of target genes by NuRD, Wdr5, Sall4, and Suz12.** *A*, Comparison of Wdr5 and NuRD target genes in mESCs. The proportional overlap of both gene data sets was visualized. The NuRD data set was derived from an overlap of Chd4, Hdac1, and Hdac2 target gene sets in mESCs. *B*, Comparison of NuRD (same as in *A*), Sall4 and Suz12 target gene sets. *C*, The shared genes in *B* were combined into a new data set (105-NuRD/Suz12; 34-NuRD/Suz12/Sall4; 342-NuRD/Sall4). This data set was reduced to genes colocalized by Wdr5. The full Sall4 and Suz12 data sets (same as in *C*) were compared with the resulting gene data set.

To explore the functional relevance of the observed interactions we investigated the genomic binding sites of the chromatin factors involved in murine ESCs. We first looked at the NuRD-Wdr5 interaction by comparing known target genes of Wdr5 and NuRD. Target loci were retrieved from published data sets generated by chromatin immunoprecipitation followed by deep sequencing (ChIP-Seq) experiments (5, 51–54). As NuRD subunits are also present in other chromatin remodeling complexes, we used the target gene overlap of Hdac1, Hdac2, and Chd4 to acquire the most accurate representation of the NuRD complex. It was apparent that 72% (1162 of 1604) of NuRD target genes were also Wdr5 targets (Fig. 6*A*). As part of SET H3K^4^ methyltransferases, Wdr5 has been shown to mark active and bivalent promoters in mESC (52, 55). Most bivalent genes are involved in developmental processes (4). We next analyzed the target gene sets for GO term enrichment. The full Wdr5 target data set is enriched in terms related to both basic cellular and developmental processes (data not shown). Interestingly, the overlapping Wdr5 and NuRD-bound set was enriched in GO terms related to development, whereas targets bound by only Wdr5 were mostly associated with basic cellular processes (supplemental Table S2). This suggests that developmental genes bound by Wdr5 are also targeted by NuRD and are thus likely to be in a bivalent chromatin state.

To put other NuRD structural observations from this study into functional context, we extended the ChIP-Seq analysis to determine target gene specificity of NuRD in association with Sall4, Wdr5, and Suz12. First, we compared the target loci of NuRD and Sall4 and performed a GO term enrichment analysis. A previous study suggested different functions for Sall4a, Sall4b, and Sall4a/b, with Sall4a suppressing developmental genes and Sall4b and Sall4a/b activating self-renewal genes (56). The Sall4-only gene cluster was enriched in terms related to basic cellular process and housekeeping genes. The NuRD-only cluster showed enrichment for housekeeping functions and some developmental processes. Interestingly, the overlap of NuRD and Sall4 targets was highly enriched in developmental functions (supplemental Table S3). The data suggests that the previously described Sall4a-mediated repression of developmental genes takes place through association with NuRD. We then determined the target genes correlation of Suz12 and Sall4, the interactors previously identified as exclusive for the NuRD I and II entities respectively. Of NuRD loci associated with either Sall4 or Suz12, only 7% were common targets, in agreement with the structural separation observed by BN-PAGE (Fig. 6*B*). Both overlapping target gene sets (NuRD-Sall4 and NuRD-Suz12) are enriched in development and differentiation-associated GO terms (supplemental Table S4). Interestingly, there is a striking difference in the enriched GO terms associated to each group. The NuRD-Sall4 gene set was associated particularly to respiratory, vasculature, and tube development. The NuRD-Suz12 set on the other hand outlined pancreatic and nervous system development. This suggests that Sall4 and Suz12 direct NuRD to different sets of developmental targets, although these did not seem to correlate with stem cell lineages. As Wdr5 was uniformly present in both entities, we aimed to illustrate the significance of Wdr5 for the interaction of NuRD with Sall4 and Suz12. We isolated genes bound by NuRD-Sall4, NuRD-Suz12, and all three proteins. The resulting target gene set was filtered for Wdr5 target loci. We then visualized the overlap of the acquired gene set with Suz12 and Sall4. 74% of previously identified NuRD-Sall4-Suz12 genes are bound by Wdr5 (Fig. 6*C*). This is suggestive of cobinding of Wdr5 at NuRD-Sall4 and NuRD-Suz12 loci.

In summary, our results confirm that NuRD interacts closely with Wdr5 at developmental loci but not housekeeping genes. NuRD target loci partially overlap with Suz12 and Sall4 targets, with a clear distinction between both interactors, similar to the clear structural separation pattern observed after BN-PAGE. Wdr5 also binds at the majority of these Suz12 and Sall4 targets.

## DISCUSSION

In this study, we used blue native PAGE followed by LC-MS/MS and protein correlation profiling (PCP) to resolve multiple protein complexes within the interactome of Mta2, a core subunit of the NuRD chromatin remodeling complex. BN-PAGE resolves two NuRD entities with differing topology, which we have termed I and II. We show that only NuRD II associates with cell cycle regulator Cdk2ap1 and transcription factor Sall4, and that this interaction is specific to isoform Sall4a. Our data suggests that, in contrast, NuRD I associates with Suz12, a subunit of the PRC2 complex. Moreover, PCP has also allowed us to identify a novel interaction with Wdr5, a core regulatory subunit of MLL methyltransferase complexes. In addition to NuRD entities, BN-PAGE resolved other interactors of Mta2, *e.g.* karyopherin Kpna2, which might be involved in the nuclear transport of Mta2. Here we have focused on interactions within NuRD and with other chromatin binding factors.

The NuRD complex plays a central role in the tight regulation of gene expression programs underlying pluripotency and differentiation of embryonic stem cells (1, 6). Lack of NuRD activity in ESCs leads to increased gene expression, failure to differentiate properly and severe developmental defects (12, 57, 58). It has been proposed that in ESCs NuRD functions as a transcriptional modulator rather than a strict silencer, at least in part by abolishing the H3K^27^ac activation mark and recruiting the Polycomb repressive complex (PRC2) to permit H3K^27^ methylation and possibly through several other mechanisms (1, 5, 7, 51). These and other studies suggest a very elaborate function of the NuRD complex in a multitude of epigenetic regulatory processes, mediated via the association to sequence-specific transcription factors or other chromatin remodeling complexes (1, 5, 51, 59). The existence of two distinct NuRD entities containing different binding partners, as suggested by our BN-PAGE migration profiles, supports this notion and might explain this functional complexity.

The low-mass NuRD I identified here most likely corresponds to NuRD-PRC2 suppressor activity at bivalent promoters (5), as a significant peak of the core subunit Suz12 was identified partially overlapping with NuRD I. We have not been able to confirm an interaction between Suz12 and NuRD subunits by co-immunoprecipitation, as others before us, suggesting that the comigration we observe might reflect co-occupancy rather than direct association (5).

In contrast, only NuRD II contained Cdk2ap1, a less-well known NuRD subunit that is required for differentiation (47–49), and Sall4, a transcription factor essential for maintaining stem cell pluripotency (60–62), that has also been reported to interact with NuRD (14, 50, 63). The functional separation between the two NuRD entities was further reinforced by a minimal target gene overlap between NuRD-Suz12 and NuRD-Sall4 target gene sets. Sall4 was originally described as a transcriptional repressor; however, further investigation suggested a function in pluripotency gene activation (56, 64, 65). Alternative splicing of *Sall4* yields two isoforms, namely Sall4a and Sall4b, which can form homodimers and a Sall4a/b heterodimer (56). In ESCs these have predominantly differing genomic binding sites and associate with different epigenetic marks: Sall4a targets are repressed developmental genes whereas Sall4b and Sall4a/b targets are active pluripotency genes (56). To mediate such distinct functions, it has previously been proposed that Sall4 isoforms associate with different chromatin remodeling complexes (56, 66). Consistently with this theory, we show that the NuRD complex interacts specifically with Sall4a and not Sall4b. Previous studies linking NuRD to Sall4 function did not make such distinction. GO term enrichment analysis of genes bound by both NuRD and Sall4 is in agreement with such trend. These loci, which we show correspond to Sall4a, were predominantly associated to developmental processes. Our data might therefore explain why only Sall4a-targeted genes are subject to repression. Interestingly, Chd4 seems more abundant in NuRD II than in NuRD I. Chd4 displays high affinity toward H3K^9^ac, an epigenetic mark that prevents H3K^4^me3 demethylation (67). Lsd1, the demethylase responsible for the removal of the H3K^4^me3 activation mark, associates with NuRD subunits on active genes in ESCs and is believed to be involved in enhancer silencing upon differentiation (51). Lsd1 and Sall4 have also been shown to occupy identical binding sites on hematopoietic regulatory genes, and lack of Lsd1 catalytic activity results in abrogation of the repressive Sall4 function (66). The disproportional abundance of Chd4 in NuRD II could provide a means of bringing together the repressor activities of Sall4a, NuRD, and Lsd1 at active genes in ESCs in preparation for silencing when differentiation takes place.

The migration profile of Wdr5 displayed two peaks that correlated perfectly with NuRD I and II, identifying a novel interaction. Close examination of data from a recent HDAC interaction study revealed that Wdr5 copurified with Hdac1 and Hdac2, but the interaction was not validated further (68). Other studies looking at NuRD stoichiometry have not identified this (14, 15). We confirmed here that Wdr5 is a *bona fide* NuRD interacting protein. The biochemical observations suggest that this interaction is strong and very close, and in this respect Wdr5 behaves like a NuRD subunit. In fact, our analysis of ChIP-Seq data sets showed that the majority of NuRD targets are also bound by Wdr5. The robust interaction of Wdr5 with NuRD suggests direct binding, rather than co-occurrence at promoters.

The Wdr5-Rbbp5-Ash2l core regulatory complex is critical for the structural integrity and catalytic activity of SET/Trithorax complexes catalyzing H3K^4^ methylation, which is essential for active transcription (45, 69, 70). Wdr5 initiates the interaction with H3 histone tails, an obligatory step for the assembly of remaining core subunits on H3K^4^me2 (55). Despite the absence of other MLL complex subunits in this data set, previous studies in our laboratory have identified Rbbp5, Ash2l, and Mll2 as Mta2-associated proteins (M. Pardo, unpublished results). This might suggest that full association of both complexes is not sufficiently stable to withstand the BN-PAGE separation. Alternatively, Wdr5 could specifically have a novel role in the NuRD complex on its own. Indeed, Wdr5 has been proposed to function as a “presenter” assisting substrate binding and selection for histone modification (70). In ESCs, Mll2 marks transcriptional start sites at bivalent promoters, with subsequent methylation possibly catalyzed by a positively reinforcing mechanism involving Mll2 and Set1C (71). Accordingly, we show that genes involved in developmental processes are highly represented only in the NuRD- and Wdr5-bound target set, but not in Wdr5-only targets. We suggest that following initial H3K^4^ methylation at the TSS, binding of NuRD to Wdr5 at further sites could prevent the association of Mll2, preventing the reinforcement of activation. Another study has shown that Wdr5 associates with Hdac3 to recruit methyltransferases for mesenchymal gene activation, whereas Hdac3 alone represses epithelial gene expression (72). Further investigation is required to elucidate the nature and role of the Wdr5-NuRD interaction.

In conclusion, we show that BN-PAGE combined with protein identification by mass spectrometry and protein correlation profiling provides a powerful technique for studying the topology of purified nuclear multiprotein complexes. This is to our knowledge the first study to combine this strategy with affinity purification to understand how individual proteins are organized in multiple complexes. This approach should be amenable to explore the topology of other interactomes. We identify a novel interaction of the NuRD chromatin remodeling complex with Wdr5. Furthermore, we uncover two distinct assemblies of the NuRD complex in mouse embryonic stem cells that associate with different partners, which might help explain its intricate role in the regulation of pluripotency and differentiation.

## Supplementary Material

Supplemental Data

## Footnotes

^1^ The abbreviations used are:

NuRD: Nucleosome Remodeling and Deacetylase complex
AP: affinity purification
BAC: bacterial artificial chromosome
BN-PAGE: blue native polyacrylamide gel electrophoresis
CBB: Coomassie Brilliant Blue G-250
ChIP-seq: chromatin immunoprecipitation followed by deep sequencing
ESC: embryonic stem cells
iBAQ: intensity based absolute quantification
LC: liquid chromatography
MS/MS: tandem mass spectrometry
PCP: protein correlation profiling
PES: polyethersulfone
PRC2: Polycomb repressive complex 2
PVDF: polyvinylidene difluoride
SEC: size exclusion chromatography.
