# Supplementary material for: Characterization of Two Distinct Nucleosome Remodeling and Deacetylase (NuRD) Complex Assemblies in Embryonic Stem Cells
Source: Mol Cell Proteomics. 2015 Dec 29;15(3):878–91. doi: 10.1074/mcp.M115.053207 (PMC4813707; doi:10.1074/mcp.M115.053207)
Supplement: Supplemental Data [file supp_15_3_878__index.html]

Characterisation of two distinct Nucleosome Remodelling and Deacetylase Complex (NuRD) assemblies in embryonic stem cells — Characterization of Two Distinct Nucleosome Remodeling and Deacetylase (NuRD) Complex Assemblies in Embryonic Stem Cells — Two Distinct NuRD Assemblies in Embryonic Stem Cells — Supplemental Data 

# Characterization of Two Distinct Nucleosome Remodeling and Deacetylase (NuRD) Complex Assemblies in Embryonic Stem Cells

## Supplemental Data

- Supplementary Figure S1 (.pdf, 214 KB) - Amino acid sequence of the FTAP2 tag
- Supplementary Figure S2 (.pdf, 254 KB) - Reproducibility of Blue Native PAGE migration profiles
- Supplementary Figure S3 (.pdf, 300 KB) - Individual BN-PAGE migration profiles of NuRD core subunits
- Supplementary Figure S4 (.pdf, 385 KB) - Correlation of core NuRD subunit migration profiles with the bait protein Mta2
- Supplementary Figure S5 (.pdf, 327 KB) - Size exclusion chromatograph of ESC whole cell lysate
- Supplementary Table S1 (.xlsx, 743 KB) - Lists of proteins identified and quantitated in four replicate experiments
- Supplementary Table S2 (.pdf, 195 KB) - GO Biological process term enrichment for NuRD and Wdr5 targets
- Supplementary Table S3 (.pdf, 205 KB) - GO Biological process term enrichment for NuRD and Sall4
- Supplementary Table S4 (.xlsx, 29 KB) - GO Biological process term enrichment for NuRD-Sall4 and NuRD-Suz12 target gene sets
