# Supplementary material for: Characterization of Two Distinct Nucleosome Remodeling and Deacetylase (NuRD) Complex Assemblies in Embryonic Stem Cells
Source: Mol Cell Proteomics. 2015 Dec 29;15(3):878–91. doi: 10.1074/mcp.M115.053207 (PMC4813707; doi:10.1074/mcp.M115.053207)
Supplement: Supplemental Data [file 10.1074_M115.053207_mcp.M115.053207-1.pdf]

GSMEKRRWKKNFIAVSAANRFKKISSSGALDYDIPTTAS
ENLYFQGELAIPTTENLYFQSGELDYKDHDGDYKDHDID
YKDDDDKCTCCIAQDKNISSRTVKLSAYINSNTRGVMEG
RGSLLTCGDVEENP

CBP 2 x Tev 3 x Flag

Supplementary Figure S1. Amino acid sequence of the FTAP2 tag. The FTAP2 tag contains the 3x FLAG peptide sequence, 2x TEV protease cleavage sites and the calmodulin binding peptide (CBP).
