# Supplementary material for: Characterization of Two Distinct Nucleosome Remodeling and Deacetylase (NuRD) Complex Assemblies in Embryonic Stem Cells
Source: Mol Cell Proteomics. 2015 Dec 29;15(3):878–91. doi: 10.1074/mcp.M115.053207 (PMC4813707; doi:10.1074/mcp.M115.053207)
Supplement: Supplemental Data [file 10.1074_M115.053207_mcp.M115.053207-4.pdf]

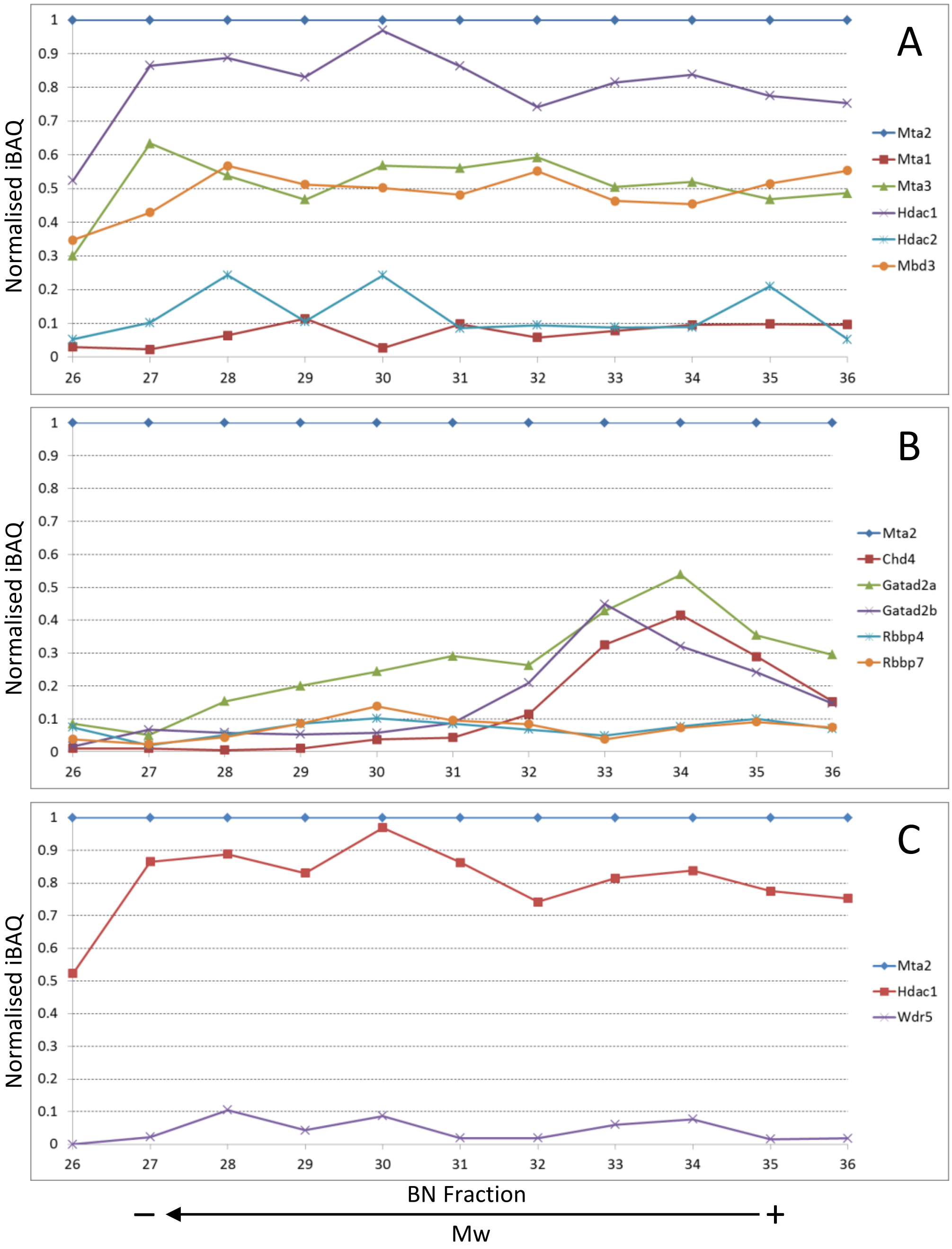

Supplementary Figure S4. Correlation of core NuRD subunit migration profiles with the bait protein Mta2. iBAQ values were derived for NuRD core subunits and Wdr5 in each fraction and normalised to the bait protein Mta2. The analysis was performed in a representative benzonase-treated replicate. A, Mta1, Mta2, Mta3, Hdac1-2, Mbd3. B, Chd4, Gatad2a, Gatad2b, Rbbp4 , Rbbp7. C, Wdr5, with Hdac1 and Mta2 as reference.
