# Supplementary material for: Characterization of Two Distinct Nucleosome Remodeling and Deacetylase (NuRD) Complex Assemblies in Embryonic Stem Cells
Source: Mol Cell Proteomics. 2015 Dec 29;15(3):878–91. doi: 10.1074/mcp.M115.053207 (PMC4813707; doi:10.1074/mcp.M115.053207)
Supplement: Supplemental Data [file 10.1074_M115.053207_mcp.M115.053207-5.pdf]

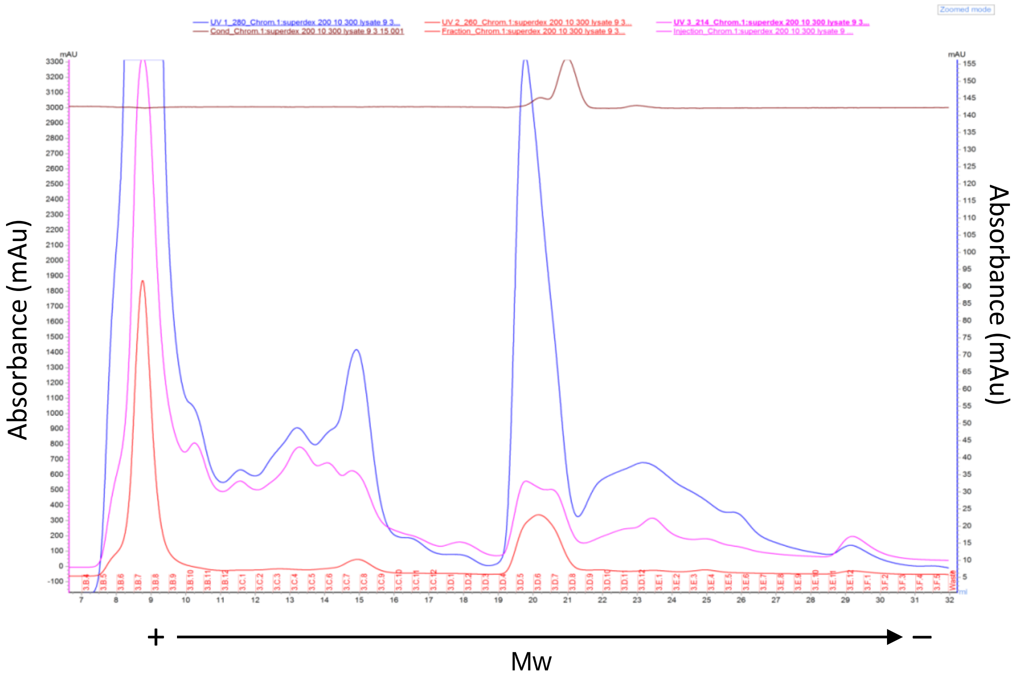

Supplementary Figure S5. Size exclusion chromatograph of ESC whole cell lysate. UV chromatographs showing the elution profile of mouse ESC whole cell lysate from a Superdex 200 10/300 size exclusion chromatography column. Readings at 260nm (DNA), 280nm (aromatic amino acid side chains) and 214nm (peptide bond) and fractions collected (x-axis) are shown. The elution range of most proteins spans until fraction 3D8.
