# Supplementary material for: Characterization of Two Distinct Nucleosome Remodeling and Deacetylase (NuRD) Complex Assemblies in Embryonic Stem Cells
Source: Mol Cell Proteomics. 2015 Dec 29;15(3):878–91. doi: 10.1074/mcp.M115.053207 (PMC4813707; doi:10.1074/mcp.M115.053207)
Supplement: Supplemental Data [file 10.1074_M115.053207_mcp.M115.053207-2.pdf]

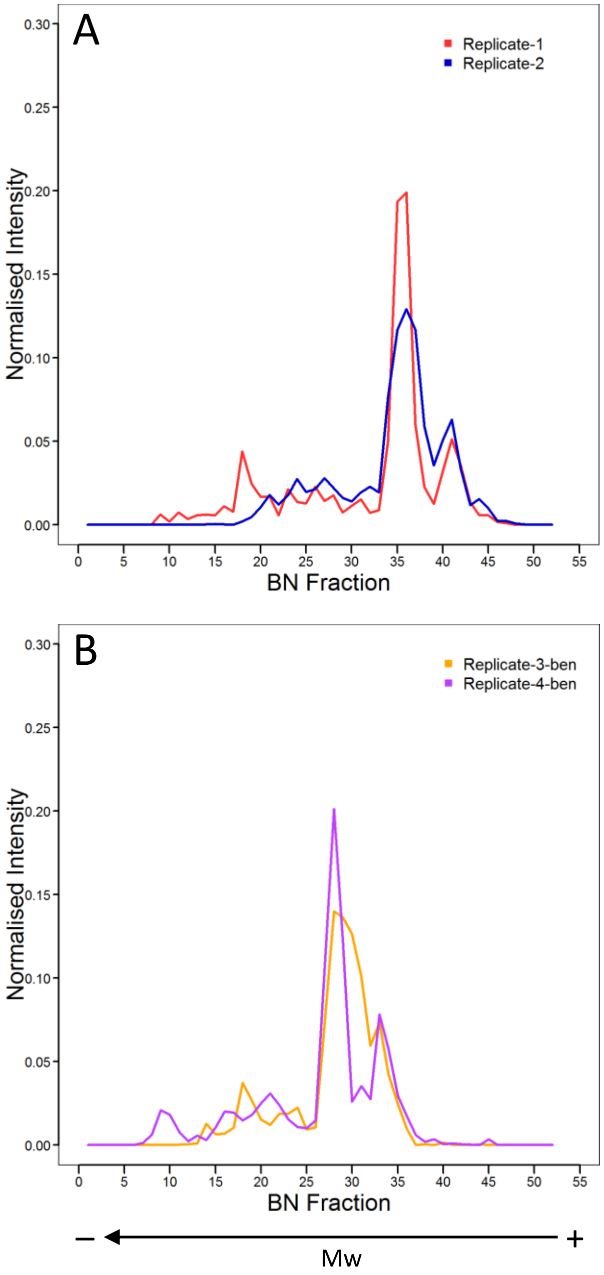

Supplementary Figure S2. Reproducibility of Blue Native PAGE migration profiles. A, Mta2 migration profiles in two independent replicate experiments. The profiles were aligned using the fraction with the highest intensity for Mta2 to allow for cutting differences between experiments. BN-PAGE fractions were numbered from the first fraction of the left-most profile. B, Replicates 3 and 4 had benzonase added during the affinity purification.
