# Supplementary material for: Characterization of Two Distinct Nucleosome Remodeling and Deacetylase (NuRD) Complex Assemblies in Embryonic Stem Cells
Source: Mol Cell Proteomics. 2015 Dec 29;15(3):878–91. doi: 10.1074/mcp.M115.053207 (PMC4813707; doi:10.1074/mcp.M115.053207)
Supplement: Supplemental Data [file 10.1074_M115.053207_mcp.M115.053207-7.pdf]

Supplementary Table S2. GO Biological process term enrichment for NuRD and Wdr5 targets. The enrichment is shown for Wdr5-only, NuRD-only and common NuRD-Wdr5 targets.

Wdr5	P-Value	Bonferroni
**Term**	**P-Value**	**Bonferroni**
GO:0044237~cellular metabolic process	3.21E-130	1.75E-126
GO:0044260~cellular macromolecule metabolic process	1.19E-104	6.48E-101
GO:0044238~primary metabolic process	2.01E-99	1.10E-95
GO:0008152~metabolic process	2.97E-95	1.62E-91
GO:0043170~macromolecule metabolic process	6.06E-84	3.31E-80
GO:0034641~cellular nitrogen compound metabolic process	1.39E-59	7.58E-56
GO:0006807~nitrogen compound metabolic process	5.79E-57	3.16E-53
GO:0006139~nucleobase, nucleoside, nucleotide and nucleic acid metabolic process	1.44E-55	7.86E-52
GO:0044267~cellular protein metabolic process	9.08E-50	4.96E-46
GO:0010467~gene expression	1.94E-48	1.06E-44
GO:0044249~cellular biosynthetic process	8.56E-44	4.67E-40
GO:0009058~biosynthetic process	1.02E-43	5.59E-40
GO:0009059~macromolecule biosynthetic process	7.44E-37	4.06E-33
GO:0034645~cellular macromolecule biosynthetic process	1.32E-36	7.18E-33
GO:0016070~RNA metabolic process	6.89E-33	3.76E-29
GO:0019538~protein metabolic process	3.98E-30	2.17E-26
GO:0006396~RNA processing	1.93E-28	1.05E-24
GO:0007049~cell cycle	1.46E-23	7.98E-20
GO:0043412~biopolymer modification	1.49E-21	8.12E-18
GO:0006996~organelle organization	3.21E-21	1.75E-17

NuRD	P-Value	Bonferroni
**Term**	**P-Value**	**Bonferroni**
GO:0009653~anatomical structure morphogenesis	6.52E-05	0.08565084
GO:0032502~developmental process	1.09E-04	0.13859055
GO:0003002~regionalization	1.37E-04	0.17169145
GO:0007275~multicellular organismal development	2.84E-04	0.32288213
GO:0007389~pattern specification process	4.21E-04	0.43895875
GO:0048513~organ development	5.53E-04	0.53211942
GO:0048856~anatomical structure development	6.85E-04	0.61008599
GO:0006357~regulation of transcription from RNA polymerase II promoter	0.0014514	0.86407929
GO:0050793~regulation of developmental process	0.0015238	0.87696747
GO:0048731~system development	0.0019426	0.9308638

Wdr5 - NuRD overlap	P-Value	Bonferroni
**Term**	**P-Value**	**Bonferroni**
GO:0009987~cellular process	1.50E-31	4.65E-28
GO:0032502~developmental process	8.40E-25	2.60E-21
GO:0007275~multicellular organismal development	9.23E-23	2.86E-19
GO:0048856~anatomical structure development	7.53E-21	2.33E-17
GO:0048519~negative regulation of biological process	2.73E-20	8.46E-17
GO:0048523~negative regulation of cellular process	3.48E-20	1.08E-16
GO:0048731~system development	6.12E-20	1.90E-16
GO:0016043~cellular component organization	1.53E-18	4.75E-15
GO:0048522~positive regulation of cellular process	3.37E-18	1.05E-14
GO:0048518~positive regulation of biological process	2.73E-17	8.47E-14
GO:0048869~cellular developmental process	2.14E-16	6.88E-13
GO:0009653~anatomical structure morphogenesis	5.36E-16	1.72E-12
GO:0030154~cell differentiation	1.39E-15	4.47E-12
GO:0048513~organ development	4.42E-15	1.38E-11
GO:0031323~regulation of cellular metabolic process	1.26E-14	3.92E-11
