# Supplementary material for: Characterization of Two Distinct Nucleosome Remodeling and Deacetylase (NuRD) Complex Assemblies in Embryonic Stem Cells
Source: Mol Cell Proteomics. 2015 Dec 29;15(3):878–91. doi: 10.1074/mcp.M115.053207 (PMC4813707; doi:10.1074/mcp.M115.053207)
Supplement: Supplemental Data [file 10.1074_M115.053207_mcp.M115.053207-8.pdf]

Supplementary Table S3. GO Biological process term enrichment for NuRD and Sall4. Enriched terms were clustered and clusters with the highest enrichment score are shown. NuRD only, Sall4 only and common NuRD-SAll4 target gene GO term enrichment is shown.

Sall4	PValue	Bonferroni
Enrichment Score: 4.114761390950519		
**Term**	**PValue**	**Bonferroni**
GO:0009987~cellular process	1.219E-06	0.002114408
GO:0080090~regulation of primary metabolic process	1.495E-06	0.002592183
GO:0019222~regulation of metabolic process	2.044E-06	0.0035429
GO:0060255~regulation of macromolecule metabolic process	3.603E-06	0.006235131
GO:0044260~cellular macromolecule metabolic process	4.853E-06	0.008388892
GO:0019219~regulation of nucleobase, nucleoside, nucleotide and nucleic acid metabolic process	5.053E-06	0.008733313
GO:0010468~regulation of gene expression	5.557E-06	0.009599798
GO:0045449~regulation of transcription	6.467E-06	0.011164208
GO:0051171~regulation of nitrogen compound metabolic process	7.011E-06	0.012097722
GO:0010556~regulation of macromolecule biosynthetic process	8.170E-06	0.014082748
GO:0031323~regulation of cellular metabolic process	9.790E-06	0.016851289
GO:0031326~regulation of cellular biosynthetic process	1.050E-05	0.018068114
GO:0009889~regulation of biosynthetic process	1.217E-05	0.02090386
GO:0034645~cellular macromolecule biosynthetic process	1.943E-05	0.033174109
GO:0009059~macromolecule biosynthetic process	2.210E-05	0.03763295
GO:0044249~cellular biosynthetic process	3.433E-05	0.057849489
GO:0044237~cellular metabolic process	4.540E-05	0.075797676
GO:0009058~biosynthetic process	4.662E-05	0.07774929
GO:0006350~transcription	6.750E-05	0.110579945
GO:0043170~macromolecule metabolic process	9.377E-05	0.150229934
GO:0044238~primary metabolic process	1.415E-04	0.217847832
GO:0010467~gene expression	2.475E-04	0.349274363
GO:0008152~metabolic process	5.423E-04	0.610002762
GO:0065007~biological regulation	1.806E-03	0.956630537
GO:0050789~regulation of biological process	2.932E-03	0.993885107
GO:0006139~nucleobase, nucleoside, nucleotide and nucleic acid metabolic process	5.560E-03	0.9999374
GO:0034641~cellular nitrogen compound metabolic process	7.427E-03	0.999997603
GO:0006807~nitrogen compound metabolic process	8.360E-03	0.999999531
GO:0006355~regulation of transcription, DNA-dependent	1.355E-02	1
GO:0051252~regulation of RNA metabolic process	1.754E-02	1
GO:0050794~regulation of cellular process	2.004E-02	1

NuRD	PValue	Bonferroni
Enrichment Score: 6.661604476657148		
**Term**	**PValue**	**Bonferroni**
GO:0048519~negative regulation of biological process	2.03E-13	5.67E-10
GO:0048523~negative regulation of cellular process	3.97E-13	1.11E-09
GO:0006357~regulation of transcription from RNA polymerase II promoter	4.49E-09	1.25E-05
GO:0009890~negative regulation of biosynthetic process	2.46E-07	6.86E-04
GO:0031327~negative regulation of cellular biosynthetic process	4.64E-07	0.00129392
GO:0010558~negative regulation of macromolecule biosynthetic process	5.09E-07	0.001418948
GO:0031324~negative regulation of cellular metabolic process	6.34E-07	0.001766706
GO:0009892~negative regulation of metabolic process	7.81E-07	0.00217538
GO:0010605~negative regulation of macromolecule metabolic process	1.75E-06	0.004869017
GO:0016481~negative regulation of transcription	5.05E-06	0.013984228
GO:0000122~negative regulation of transcription from RNA polymerase II promoter	5.38E-06	0.014895512
GO:0045892~negative regulation of transcription, DNA-dependent	5.82E-06	0.016096568
GO:0051253~negative regulation of RNA metabolic process	6.76E-06	0.018661387
GO:0010629~negative regulation of gene expression	9.85E-06	0.027075959
GO:0045934~negative regulation of nucleobase, nucleoside, nucleotide and nucleic acid metabolic process	1.03E-05	0.0283823
GO:0051172~negative regulation of nitrogen compound metabolic process	1.30E-05	0.03572734
Enrichment Score: 6.166335196357349		
**Term**	**PValue**	**Bonferroni**
GO:0032502~developmental process	7.62E-10	2.12E-06
GO:0007275~multicellular organismal development	1.14E-08	3.17E-05
GO:0048856~anatomical structure development	1.29E-08	3.60E-05
GO:0048731~system development	4.94E-08	1.38E-04
GO:0048869~cellular developmental process	1.02E-07	2.85E-04

GO:0009653~anatomical structure morphogenesis	2.10E-07	5.84E-04
GO:0030154~cell differentiation	8.87E-07	0.00246882
GO:0048513~organ development	3.63E-06	0.010072738
GO:0007399~nervous system development	1.08E-04	0.260500788
GO:0032501~multicellular organismal process	0.52435	1

Sall4 - NuRD overlap	PValue	Bonferroni
Enrichment Score: 6.524009391117976		
**Term**	**PValue**	**Bonferroni**
GO:0009653~anatomical structure morphogenesis	1.60E-09	2.62E-06
GO:0032502~developmental process	9.93E-09	1.63E-05
GO:0007275~multicellular organismal development	1.11E-08	1.81E-05
GO:0048731~system development	2.23E-08	3.66E-05
GO:0048856~anatomical structure development	2.84E-08	4.65E-05
GO:0009790~embryonic development	8.03E-08	1.31E-04
GO:0048513~organ development	1.90E-07	3.10E-04
GO:0048869~cellular developmental process	6.78E-06	0.011037281
GO:0030154~cell differentiation	2.06E-05	0.03309081
GO:0032501~multicellular organismal process	0.024306102	1
Enrichment Score: 5.761284204688291		
**Term**	**PValue**	**Bonferroni**
GO:0009653~anatomical structure morphogenesis	1.60E-09	2.62E-06
GO:0048646~anatomical structure formation involved in morphogenesis	2.92E-08	4.78E-05
GO:0009790~embryonic development	8.03E-08	1.31E-04
GO:0048598~embryonic morphogenesis	1.51E-06	0.002466881
GO:0043009~chordate embryonic development	1.77E-05	0.028594254
GO:0009792~embryonic development ending in birth or egg hatching	2.04E-05	0.032789061
GO:0001701~in utero embryonic development	7.76E-05	0.119320201
GO:0007389~pattern specification process	5.12E-04	0.567647051
